# Ultrashort vortex from a Gaussian pulse – An achromatic-interferometric approach

**DOI:** 10.1038/s41598-017-02613-3

**Published:** 2017-05-24

**Authors:** Dinesh N. Naik, Nabil A. Saad, D. Narayana Rao, Nirmal K. Viswanathan

**Affiliations:** School of Physics, University of Hyderabad, Hyderabad, 500046 India

## Abstract

The more than a century old Sagnac interferometer is put to first of its kind use to generate an achromatic single-charge vortex equivalent to a Laguerre-Gaussian beam possessing orbital angular momentum (OAM). The interference of counter-propagating polychromatic Gaussian beams of beam waist ω_λ_ with correlated linear phase (*ϕ*_*0*_ ≥ 0.025 λ) and lateral shear (*y*_*0*_ ≥ 0.05 ω_λ_) in orthogonal directions is shown to create a vortex phase distribution around the null interference. Using a wavelength-tunable continuous-wave laser the entire range of visible wavelengths is shown to satisfy the condition for vortex generation to achieve a highly stable white-light vortex with excellent propagation integrity. The application capablitiy of the proposed scheme is demonstrated by generating ultrashort optical vortex pulses, its nonlinear frequency conversion and transforming them to vector pulses. We believe that our scheme for generating robust achromatic vortex (implemented with only mirrors and a beam-splitter) pulses in the femtosecond regime, with no conceivable spectral-temporal range and peak-power limitations, can have significant advantages for a variety of applications.

## Introduction

Invented more than a century ago, the Sagnac interferometer (SI) with its common-path configuration providing immunity against surrounding vibrations and turbulences upon perfect alignment, utilizes time of flight difference between clock-wise and counter clock-wise propagating beams, to measure the fringe shift proportional to the angular velocity of the rotating stage^1, 2^. The SI is the back bone of modern day gyroscopes for inertial, non-inertial and global navigation systems^3, 4^ with capabilities to measure angle of rotation up to parts-per-billion (ppb) or better sensitivity^5, 6^. The recently proposed zero-area Sagnac interferometer for gravitational wave detection showed that its rotational sensitivity can be tuned as well^7, 8^. In addition, unlike dual-path interferometer, its spectral insensitivity perceived through immediate observation of white-light fringes upon alignment is tailored for its use as Fourier transform spectrometer^9^. With tunable sensitivity against surrounding vibrations and turbulences and spectral diversity, the versatile SI interferometer is seen to be an ideal candidate for the proposed, first of its kind use to generate an achromatic single-charge vortex equivalent to a Laguerre-Gaussian beam from a polychromatic Gaussian beam.

In years that followed Nye and Berry’s seminal work^10, 11^, the generation of optical vortex beams, with embedded phase singularity is demonstrated via exciting vortex modes in laser cavity^12^ and their generation outside the laser cavity using astigmatic mode converters^13–15^, spiral phase plate^16^, computer generated holograms and diffractive optical elements displayed on spatial light modulators^17, 18^, helical mirrors^19^ and more recently using optical fibers^20^, birefringent z-cut uniaxial crystals^21^, q-plates^22^ and two-beam interference where the contributions come from both amplitude and phase difference between the superposing optical fields^23–27^ have demonstrated the sheer diversity of techniques capable of introducing the phase singularity in optical beams. The knowledge that the light beams having phase singularities carries and imparts orbital angular momentum (OAM)^28–30^ lead to their widespread applications in optical trapping, manipulation of atoms and micro particles, laser ablation and surface structuring^31–33^. The state-of-the art devices like spatial light modulators (SLMs), and the diffractive optical elements (DOEs) such as holograms have the capability to generate complex phase-structured optical beams with variable orbital angular momentum.

One of the desirable aspects for the vortex generation methods is being amenable to spectral diversity^34–36^. The generation of achromatic vortex has attracted a lot of research interest in recent years due to their potential in applications with broadband, intense, ultrashort laser pulses. In the past decade, several different schemes are proposed for generating achromatic vortex through dispersion compensation^37–41^, by introducing a radial phase modulation for spiral phase plates^42^, via spin-to-orbit conversion through Geometric phase accumulation^21, 43–46^ and using conical glass devices exploiting total internal reflection^47–49^. However, these schemes require a dedicated optical component for the task and its fabrication process often turns out to be expensive and demanding to keep the efficiency and quality of the generated achromatic vortex at an acceptable level. Moreover, the dedicated devices that works reasonably well for visible spectrum may not be suitable for other regions of the electromagnetic spectrum.

We propose and demonstrate the generation of an achromatic single-charge vortex merely from the interference of two Gaussian beams in a Sagnac interferometer with correlated linear phase and lateral shear. The SI configuration envisioned satisfies the condition for vortex generation for the entire range of visible wavelengths, enabling realization of white-light vortex. We demonstrate the practical application of the design by generating ultrashort vortex pulses. It is worth noting that high quality ultrashort single-charge vortex pulses can now be generated elegantly using inexpensive and off-the-shelf optical components. The second harmonic generation resulting in doubling of the topological charge for the generated vortex demonstrates its suitability for nonlinear optical applications^50^. Further, we also demonstrate the application capability of the generated ultrashort vortex pulse in femtosecond regime by transforming it to achromatic vector pulses^51–58^ by designing a cylindrical vector beam generator using modified Mach-Zehnder interferometer.

## Results

**Figure 1:**
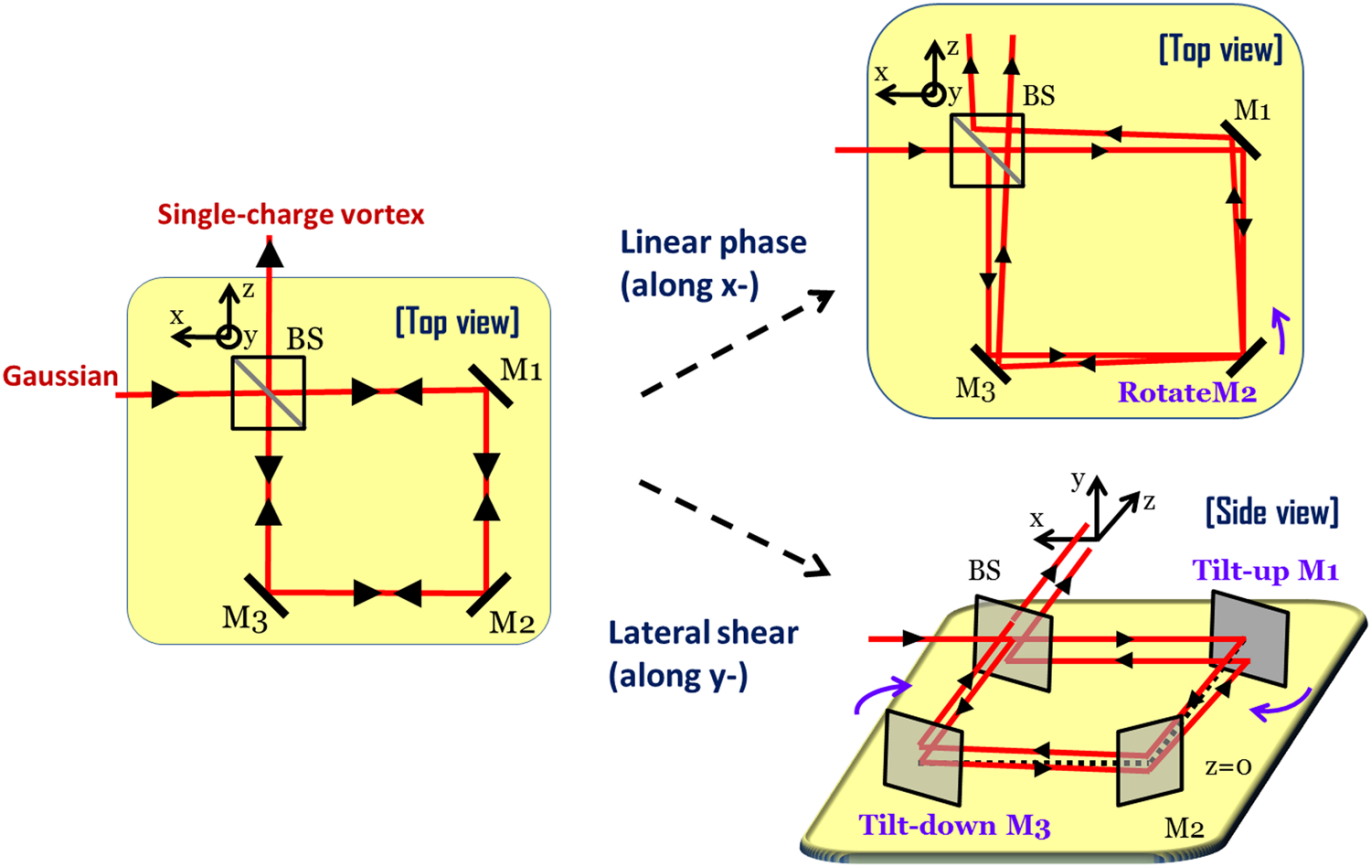
The Sagnac interferometer as single-charge vortex generator. A collimated Gaussian beam is transformed to an optical beam with single-charge vortex by introducing a linear phase difference along *x*- and lateral shear along *y*- between the interfering out-of-phase Gaussian beams.

We begin by validating the achromatic nature of the proposed Sagnac vortex generator described in Fig. 1, by experimentally generating a single-charge vortex for optical field components spanning the visible range using wavelength-tunable continuous wave laser.

As shown in Fig. 1, in a Sagnac interferometer^1–9, 59^, a controlled amount of linear phase difference with a value *ϕ*_0_ at the beam waist *ω*_λ_ corresponding to a spectral component with wavelength *λ*, is introduced between the two counter-propagating beams along *x-* by rotating mirror M_2_ by an amount α such that $\tan \,\alpha =\frac{{\varphi }_{0}{\lambda }_{0}}{{\omega }_{\lambda }}$. By tilting up the mirror M_1_ and tilting down the mirror M_3_ a required amount of lateral shear *y*_0_ for the beam along *y*- is also introduced simultaneously.

**Figure 2:**
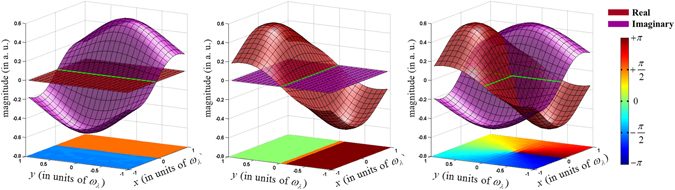
The surface plots of real and imaginary parts of the superposition of out-of-phase Gaussian beams with (**a**) linear phase difference along *x-*, (**b**) lateral shear along *y-*and (**c**) correlated linear phase along *x-* and lateral shear along *y-*. The plot at the base shows the resulting phase distribution.

The surface plots of real and imaginary parts of the superposition of out-of-phase Gaussian beams resulting from the application of the linear phase applied along *x-*, the lateral shear along *y-* and their simultaneous correlated presence are shown in Fig. 2(a–c), respectively. The crossing of the surface of imaginary part resulting from the application of linear phase with the plane of real part with zero magnitude results in a line singularity with a phase profile shown at the base of the Fig. 2(a). Similarly, the crossing of surface of real part resulting from the application of lateral shear with the plane of imaginary part with zero magnitude results in another line singularity with a phase profile shown at the base of the Fig. 2(b). It is worth noting that though the phase profiles in both the cases exhibits a jump of an amount *π* across the line singularity, they are shifted by *π/*2 with respect to each other. As represented in Fig. 2(c), it is the *‘in quadrature’* nature of the two line singularities that eventually results in formation of a point singularity having a spiral phase when the linear phase and lateral shear are introduced in orthogonal directions. The equations used in the simulations are detailed in methods section and the values used are discussed below.

### Achromatic vortex from two Gaussian beam interference

**Figure 3:**
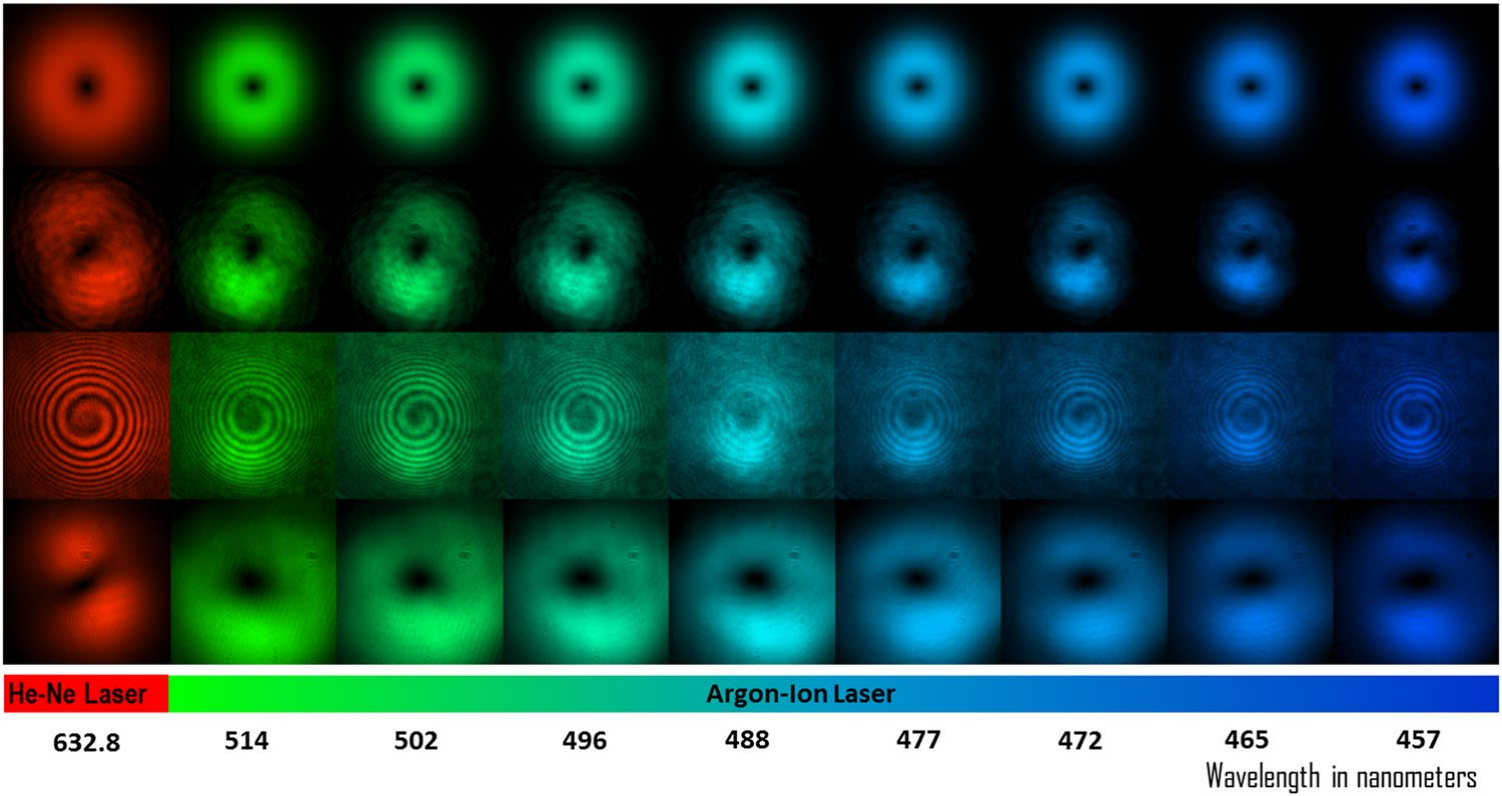
Row 1: The simulated amplitude distribution of the vortex generated for He-Ne laser and Argon-ion laser. Row 2: Experimentally recorded intensities of single charge vortex obtained merely by scanning the wavelength using He-Ne laser followed by Argon-ion laser. Row 3: The interference of the generated vortex beams with a spherical wavefront reference beam showing the spiral fringes. Row 4: the recorded intensities after a free-space propagation of 5 metres.

The simulated amplitude distribution for the single-charge vortex for the spectral component corresponding to wavelength *λ* = 632.8 *nm* of He-Ne laser and *λ* = 514 *nm*, 502 *nm* 496 *nm*, 488 *nm*, 477 *nm*, 465 *nm*, and 457 *nm* the spectral lines from an Argon-ion laser are shown in the first row of Fig. 3. A linear phase difference along *x-* having a value of *ϕ*_0_ = 0.1 in units of 2*π* and *y*_0_ ≈ 0.3*ω*_λ0_ with *ω*_λ0_ = 2.5 *mm* for central wavelength of *λ* = 545 *nm* are used for presenting the simulation results. For the rest of the wavelengths the beam waist is chosen such that, *ω*_λ_ = *pω*_λ0_ with the factor *p* attributed to the beam size scaling for different spectral components collimated from a point-like source such as output of an optical fiber. The experimentally obtained intensities of single-charge vortices corresponding to the different spectral lines recorded merely by scanning the wavelength using He-Ne laser followed by Argon-ion laser are shown in the second row of Fig. 3. The spiral interference fringes, indicating the presence of phase singularity, due to the interference of the generated vortex beams with a spherical wavefront reference beam are shown in the third row of Fig. 3. The recorded intensities of the generated vortex beam after a free-space propagation of 5 metres are shown in the fourth row of Fig. 3. The propagation stability and integrity of the generated vortex beam behaving like a Laguerre-Gaussian beam of first order are put to test when the generated vortex is used for second-harmonic generation and vector beam generation in the femtosecond pulse regime.

### Ultrashort Gaussian to vortex pulses

**Figure 4:**
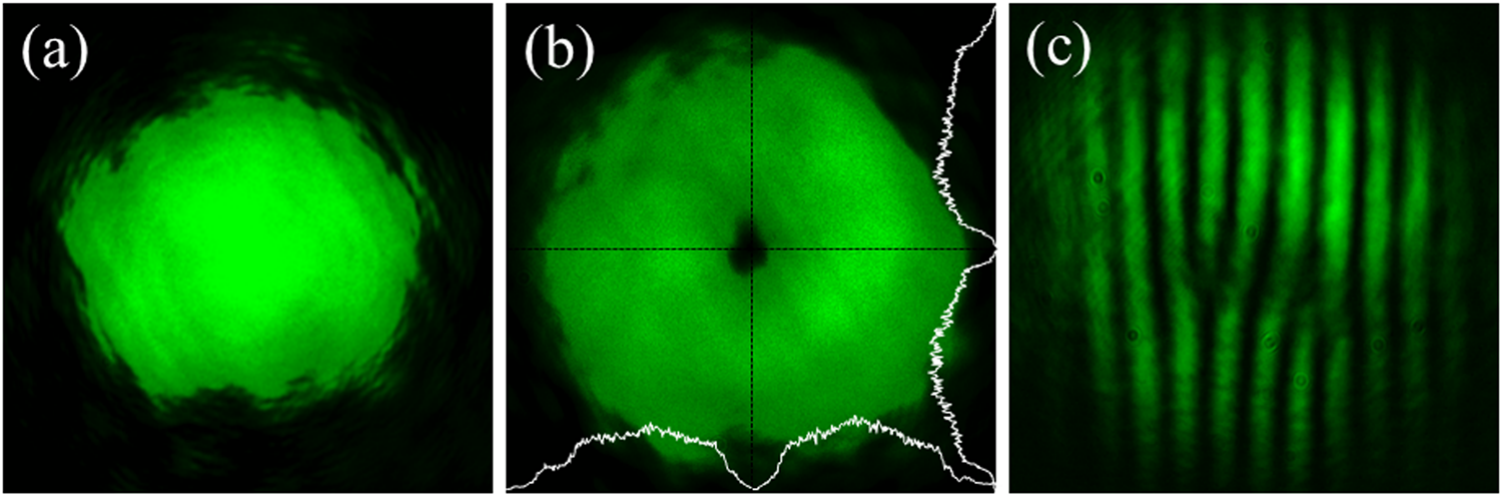
Femtosecond vortex. (**a**) Spatial intensity profile of the input femtosecond pulse at 515 nm; (**b**) the single-charge vortex generated using Sagnac achromatic vortex generator shown in Fig. 1; (**c**) the interference of the generated vortex pulse with its laterally separated tilted copy reveals the forking of fringes at the dark core of the interfering vortices.

A 300 femtosecond pulsed fiber laser having a repetition rate of 200KHz with mean wavelength 1030 nm (*SATSUMA*, *Amplitude Systemes fiber laser*) is frequency doubled to 515 nm using a BBO crystal. The spatial intensity profile of the femtosecond pulse is given in Fig. 4(a). The single charge vortex generated using the Sagnac achromatic vortex generator is shown in Fig. 4(b). The fork pattern in the fringes at the dark core of the vortex due to interference of the generated vortex pulse with its laterally separated and tilted copy confirms the presence of phase singularity.

**Figure 5:**
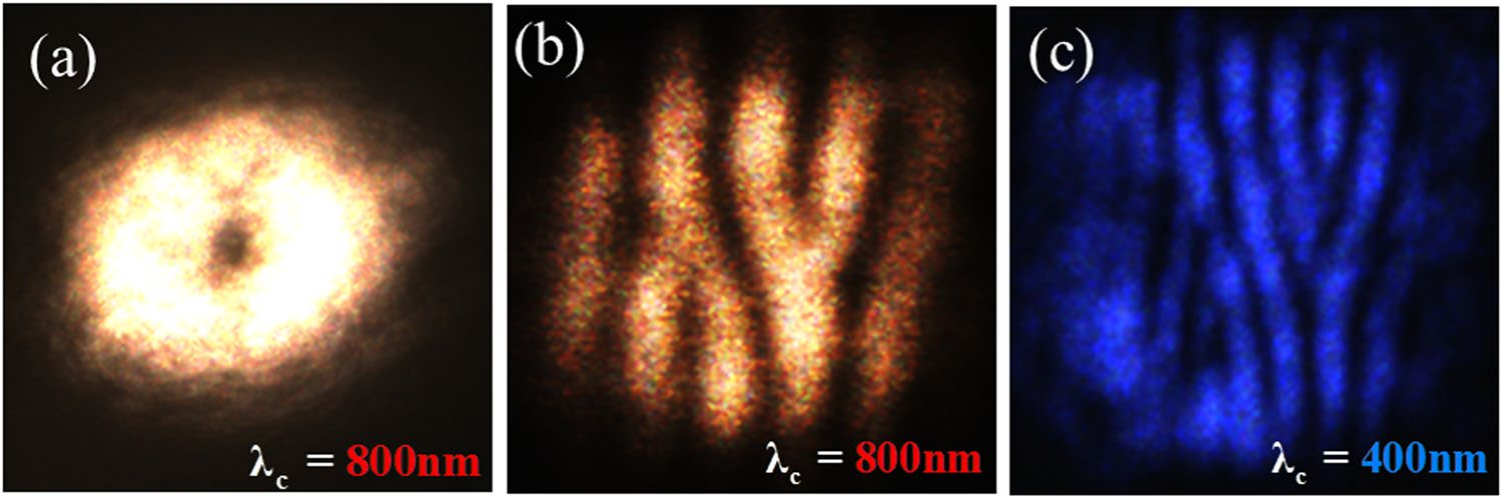
Femtosecond vortex (**a**) Single charge vortex generated using Sagnac achromatic vortex generator shown in Fig. 1. (**b**) The tilted interference of the generated vortex pulse with its laterally separated copy revealing the forking of fringes at the dark core of the interfering vortices (**c**) doubling of topological charge of the vortex through nonlinear BBO crystal.

Interchanging the sequence of second harmonic generation and vortex generation, we demonstrate the charge doubling through frequency doubling of a 800 nm and 100 fs single-charge vortex pulse (repetition rate of 1 KHz; Ti-Sapphire laser system from *Spectra Physics*). Figure 5(a) shows the intensity distribution of the generated vortex pulse and Fig. 5(b) shows the tilted interference of the vortex with its shifted copy. Introduction of the BBO crystal at the output of the Sagnac achromatic vortex generator, the forking of a fringe to three instead of two in the interference pattern shown in Fig. 5(c) confirms the charge doubling of vortices associated with second harmonic generation^50^.

### Ultrashort vortex to vector pulses

A superposition of the optical field with opposite topological charges +*l* and −*l* having orthogonal spin ${\sigma }^{+}$ and ${\sigma }^{-}$ (circular states of polarization) results in the generation of cylindrical vector beams^51–53^. In many interferometric schemes for cylindrical vector beam generation with a Gaussian beam at the input, vortex generators such as spiral phase plates are introduced inside the interferometer where the counter propagating beams acquire opposite topological charge^53–58^. To generate a vector beam from a vortex beam having a topological charge +*l*, a ‘Sagnac-like’ interferometer is recently proposed^60^. The experimental geometry used generates vector beams with a quasi-monochromatic light source, but could not be applied to ultrashort pulses due to unequal path delay and dispersion incompatibility between the interfering beams.

**Figure 6:**
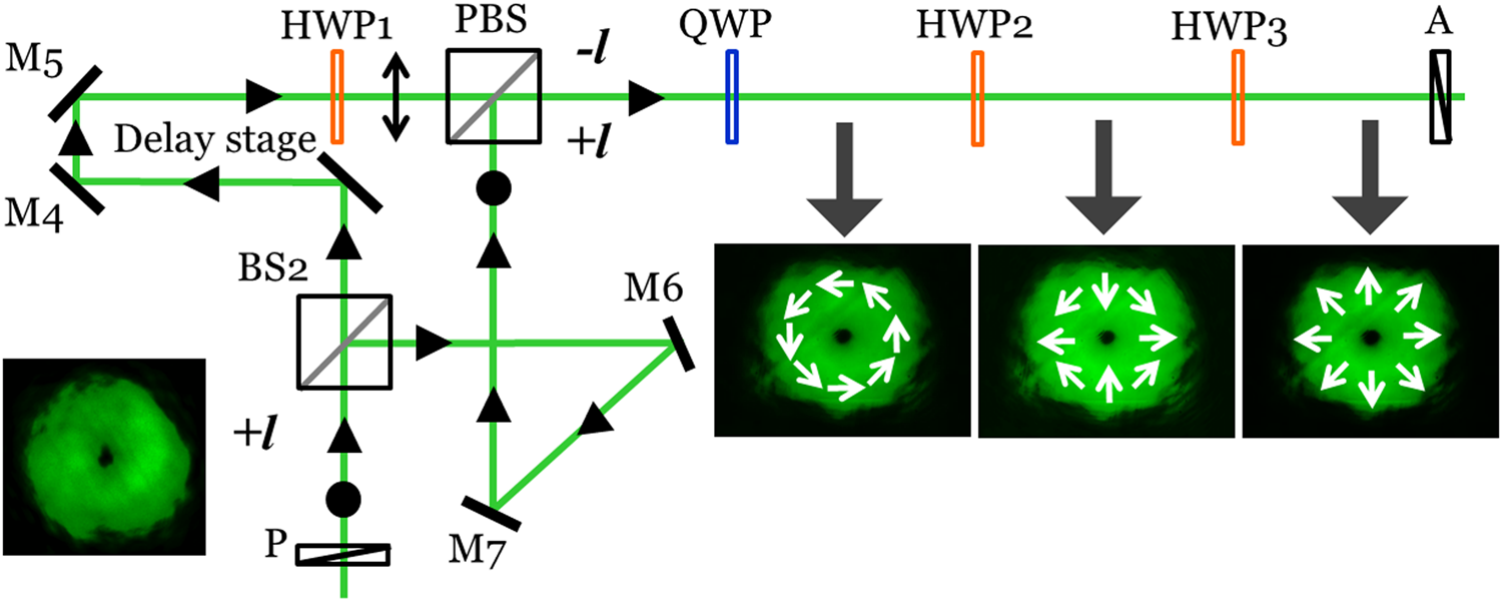
Modified Mach-Zehnder interferometer for the generation of ultrashort cylindrical vector pulses from a scalar single-charge vortex pulse.

We demonstrate the generation of ultrashort cylindrical vector pulses from a scalar single-charge vortex pulse by designing a modified Mach-Zehnder interferometer. The scalar, single-charge vortex generated using a Sagnac achromatic vortex generator with a frequency doubled, 1030 nm, 300fs laser pulse is fed to the input of the Mach-Zehnder vector beam generator shown in Fig. 6. It can be inferred that the part of the pulse split using a beam splitter (BS_2_) that travel through arm I comprising of mirrors M_4_ and M_5_ has a total of 3 reflections whereas the part of the pulse that travel through arm II comprising of mirrors M_6_ and M_7_ has a total of 4 reflections. The odd-even number of reflections ensures that the pulses have opposite topological charge at the output of the interferometer.

**Figure 7:**
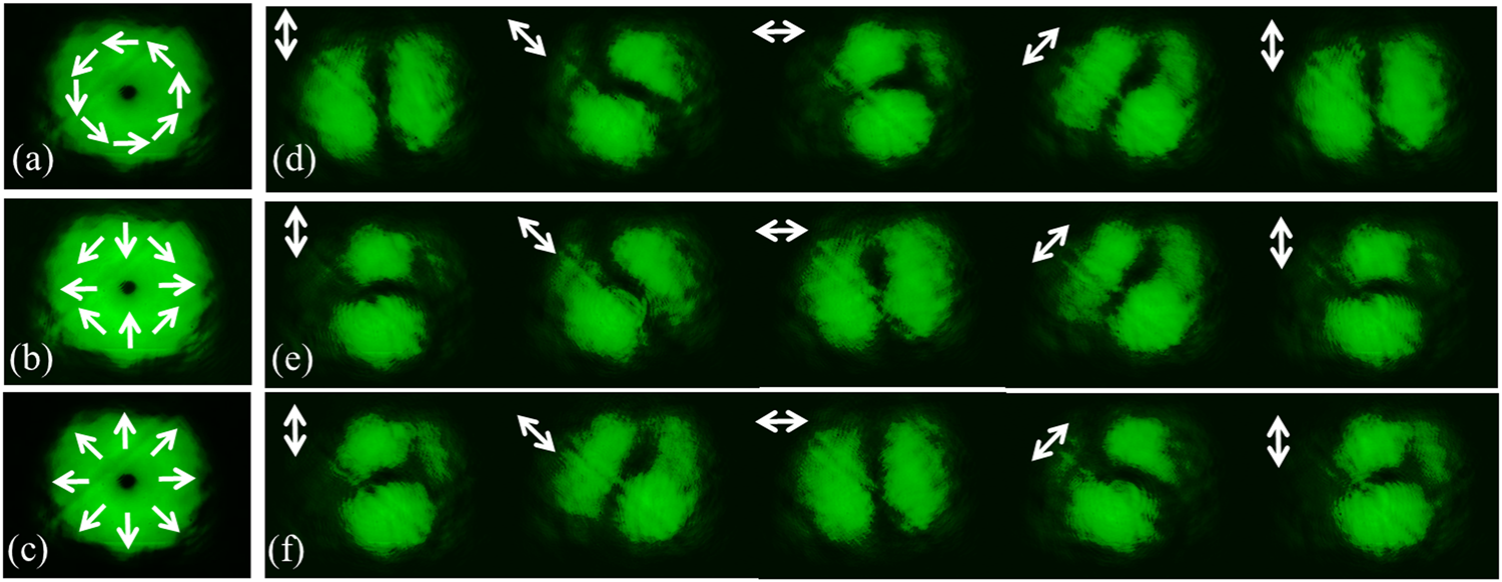
The intensity profiles of femtosecond cylindrical vector pulse. (**a**) Azimuthal (**b**) Hybrid and (**c**) Radially polarized pulses. (**d**–**f**) A rotating two-lobe intensity pattern corresponding to (**a–c**) as a function of a rotating analyser. The orientation of the axis of the analyzer is represented as double sided arrows.

Additionally, the state of polarization of the pulses are made orthogonal by introducing a half wave plate (HWP_1_) in arm I. To guarantee that they remain orthogonally polarized during their recombination and for optimum utilization of the pulse power, we used a polarizing beam splitter (PBS) at the output of the interferometer. A quarter wave plate (QWP) transforms the orthogonal linear polarizations to orthogonal spins for the pulses. As shown in Fig. 7(a–c), we observed an azimuthally polarized vector pulse after the QWP that is converted to hybrid and radially polarized pulses using half wave plates HWP_2_ with its fast axis oriented along 45° with respect to the *x-*axis and HWP_3_ oriented along 90° with respect to the *x-*axis, respectively.

We confirm the vector nature and type of the pulses by introducing a rotating analyser where a rotating two-lobe intensity pattern shown in Fig. 7(d–f) corresponding to Fig. 7(a–c) reveals the underlying spatial distribution of the polarization of the pulses.

## Discussion

Optical dispersion plays a decisive role in the generation of vortices for optical field with spectral diversity. As mentioned earlier, different approaches are designed to compensate or overcome it. In the proposed interferometric approach of introducing lateral shear and linear phase difference in orthogonal directions between superposing out-of-phase Gaussian beams, a hinge-point thus appearing plays a crucial role^61^. The source of achromatic nature of the generated vortex can be easily understood from the well-known optical white-light interferometry. The importance of zeroth-order fringe in white-light interferometry where the phase difference of all the interfering spectral components has a zero crossing to obtain a ‘white fringe’ on constructive interference or the ‘dark fringe’ on null interference with the choice of the output port of the interferometer was used to pin point the location of zero optical path difference (OPD) in optical metrology schemes^62^. From the null-interference, a correlated lateral shear and linear phase difference applied orthogonal to each other is shown to be capable of inducing a vortex around the null point for all the spectral components. The choice of Sagnac interferometer with common-path configuration right away satisfies a stable vortex pattern around zero OPD. For the chosen output of the Sagnac interferometer, the clock wise propagating Gaussian beam gets transmitted twice through beam splitter (BS) and its counterpart gets reflected twice at the BS. This asymmetry renders the output port unbalanced and satisfies the condition for out-of-phase or null interference^63^. The evenness in introducing linear phase and lateral shear between the interfering beams ensured that the hinge-point is located at *x*,*y* = 0 to generate a high-quality single-charge vortex with excellent propagation stability. The novelty of the work presented is the first of its kind use of the century-old Sagnac interferometer without any modifications or introduction of optical elements such as lenses or prisms inside the interferometer, to generate an achromatic vortex from null-interference of Gaussian beams and is therefore not compromised by the demonstrations in earlier works^25, 27^. In the proposed scheme of vortex generation using the typical Sagnac interferometer, any dispersion leading to stretching of the pulse, experienced equally by both the counter-propagating beams, does not affect the spatial profile of the generated vortex, as exemplified by the cw studies. However, the conversion of the generated achromatic vortex pulse to a vector pulse using the Mach-Zehnder interferometer requires stability of the non-commonpath interferometer and the use of achromatic wave plates. The second harmonic generation resulting in doubling of the vortex charge and the transformation of vortex pulses into vector pulses are included in our reports to substantiate that the generated vortex pulse behaves exactly like a Laguerre-Gaussian beam. We believe that our scheme for generating achromatic vortex pulses in femtosecond regime with only mirrors and beam splitter, the simplest of optical components, has an unprecedented advantage over the existing optical vortex beam generation techniques paving way for subwavelength shaping of high-power ultrashort electromagnetic pulses across the spectrum, not limited to extreme ultraviolet and X-rays^64, 65^.

## Method

### Interferometric generation of achromatic vortex from Gaussian beam

Let us assume a Gaussian spatial profile of a source of light diverging from the ouput of a fiber as

1 $${E}_{0}(\tilde{x},\tilde{y})=\tilde{A}\exp (-\frac{({\tilde{x}}^{2}+{\tilde{y}}^{2})}{{\tilde{\omega }}_{0}^{2}})$$

where $\tilde{A}$ is the maximum amplitude, ${\tilde{\omega }}_{0}$ radius of the light spot having a Gaussian profile and $\tilde{x}$ and $\tilde{y}$ are the spatial coordinates. The spectral component corresponding to the central wavelength *λ*_0_ in the light beam collimated from such a point-like source using a lens of focal length *f* can be written as

2 $${E}_{\lambda 0}(x,y)=\frac{\tilde{A}}{i{\lambda }_{0}f}\iint \exp (-\frac{({\tilde{x}}^{2}+{\tilde{y}}^{2})}{{\tilde{\omega }}_{0}^{2}})\exp [-i\frac{2\pi }{{\lambda }_{0}f}(\tilde{x}x+\tilde{y}y)]d\tilde{x}d\tilde{y}$$

We can treat the profile of *Eλ*_0_(*x*,*y*) as well a Gaussian such that

3 $${E}_{\lambda 0}(x,y)=A\exp (-\frac{({x}^{2}+{y}^{2})}{{\omega }_{{\lambda }_{0}}^{2}})$$

where *A* is the maximum amplitude, ω_*λ*0_ is the beam waist and *x* and *y* are the spatial coordinates. The field distribution corresponding to any other spectral component having wavelength *λ* = *pλ*_0_ can be written as

4 $$\begin{array}{rcl}{E}_{\lambda }(x,y) & = & \frac{A}{ip{\lambda }_{0}f}\iint \exp (-\frac{({\tilde{x}}^{2}+{\tilde{y}}^{2})}{{\tilde{\omega }}_{0}^{2}})\exp [-i\frac{2\pi }{p{\lambda }_{0}f}(\tilde{x}x+\tilde{y}y)]d\tilde{x}d\tilde{y}\\  & = & \frac{1}{p}{E}_{\lambda 0}(\frac{x}{p},\frac{y}{p})=\frac{A}{p}\exp (-\frac{({x}^{2}+{y}^{2})}{{p}^{2}{\omega }_{{\lambda }_{0}}^{2}})\end{array}$$

With *E*_*λ*_(*x*, *y*) as the spectral component of the collimated Gaussian beam at the input of Sagnac vortex generator described in Fig. 1. We write ${E}_{\lambda }^{+}(x,y)$ and ${E}_{\lambda }^{-}(x,y)$, a spectral component of the counter-progagating optical fields at the plane of mirror M_2_ and viewed from the output of the Sagnac interferometer as

5 $${E}_{\lambda }^{+}(x,y)=\frac{A}{2p}\exp (-\frac{({x}^{2}+{(y-{y}_{0})}^{2})}{{p}^{2}{\omega }_{{\lambda }_{0}}^{2}}+i\frac{2\pi }{p{\lambda }_{0}}(\frac{{\varphi }_{0}{\lambda }_{0}}{{\omega }_{{\lambda }_{0}}})x)$$

6 $${E}_{\lambda }^{-}(x,y)=\frac{A}{2p}\exp (-\frac{({x}^{2}+{(y+{y}_{0})}^{2})}{{p}^{2}{\omega }_{{\lambda }_{0}}^{2}}-i\frac{2\pi }{p{\lambda }_{0}}(\frac{{\varphi }_{0}{\lambda }_{0}}{{\omega }_{{\lambda }_{0}}})x+i\pi ).$$

The parameters *y*_0_ and *ϕ*_0_ controlling the lateral shear and linear phase for the two beams are described in Fig. 2. As discussed, the asymmetry in number of transmissions and reflections at the beam splitter for the counter-propagating beams offers the condition for an out-of-phase superposition of the two Gaussian beams at the chosen output of the interferometer. The optical field distribution at the output of the Sagnac interferometer can be represented as

7 $$\begin{array}{rcl}{E}_{\lambda }^{o}(x,y) & = & {E}_{\lambda }^{+}(x,y)+{E}_{\lambda }^{-}(x,y)\\  & = & \frac{A}{p}\exp (\frac{-{y}_{0}^{2}}{{p}^{2}{\omega }_{{\lambda }_{0}}^{2}})\sinh (\frac{2{y}_{0}}{{p}^{2}{\omega }_{{\lambda }_{0}}^{2}}y+i\frac{2\pi {\varphi }_{0}}{p{\omega }_{{\lambda }_{0}}}x)\exp (-\frac{({x}^{2}+{y}^{2})}{{p}^{2}{\omega }_{{\lambda }_{0}}^{2}})\end{array}.$$

Restricting the variation of the real and imaginary parts of the argument of ‘sinh’ term to the linear region of the hyperbolic sine function and sine function respectively, we can simplify Eq. (7) as

8 $${E}_{\lambda }^{o}(x,y)\approx \frac{A}{p}\exp (\frac{-{y}_{0}^{2}}{{p}^{2}{\omega }_{{\lambda }_{0}}^{2}})(\frac{2{y}_{0}}{{p}^{2}{\omega }_{{\lambda }_{0}}^{2}}y+i\frac{2\pi {\varphi }_{0}}{p{\omega }_{{\lambda }_{0}}}x)\exp (-\frac{({x}^{2}+{y}^{2})}{{p}^{2}{\omega }_{{\lambda }_{0}}^{2}}).$$

The term $(\frac{2{y}_{0}}{{p}^{2}{\omega }_{{\lambda }_{0}}^{2}}y+i\frac{2\pi {\varphi }_{0}}{p{\omega }_{{\lambda }_{0}}}x)$ could describe a canonical single-charge vortex having a uniform phase variation as a function of azimuthal coordinate $\phi ={\tan }^{-1}(y/x)$ under a condition that ${y}_{0}/(p{\omega }_{{\lambda }_{0}})=\pi {\varphi }_{0}$. The source of spiral phase, the term having the form $(ay+ibx)$ with *a* and *b* as coefficients, in the superposition of every spectral component highlights the geometric nature of the vortex generation and its associated achromaticity.

**Figure 8:**
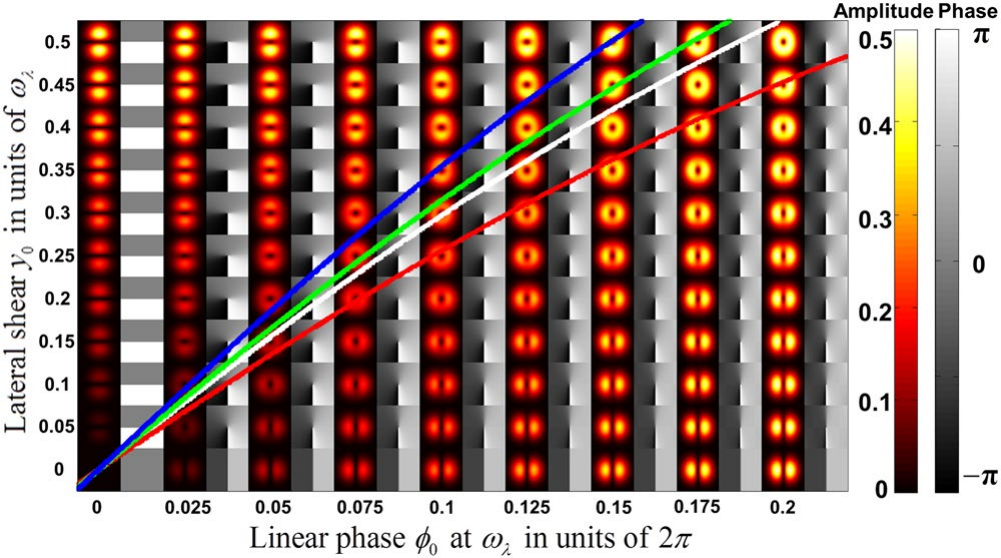
The parameter plot for generation of single-charge vortex. The white line describes the locus of single-charge vortex with symmetric core for central wavelength of *λ*_0_ = 545 *nm*. The red, green and blue lines describe the locus of the single-charge vortices that could be generated for *λ* = 632.8 *nm* of He-Ne laser and *λ* = 514 *nm* and *λ* = 457 *nm* from Argon-ion laser under the parameters set for generating symmetric core vortex for the central wavelength.

To understand the influence of difference in the ratio of lateral shear *y*_0_ to the beam waist *ω*_*λ*_ of different spectral components on the symmetry of the generated single-charge vortex, a parameter plot of lateral shear *y*_0_(in units of *ω*_*λ*_) against linearly increasing phase *ϕ*_0_ at *ω*_*λ*_(in units of 2*π*) is shown in Fig. 8. The white line represents the locus of points satisfying the condition, $\sinh (2{y}_{0}/{\omega }_{{\lambda }_{0}})=2\pi {\varphi }_{0}$ on which the generated vortices for the central wavelength of *λ*_0_ = 545 *nm* are canonical with a symmetric dark core. For the same parameter settings of *y*_0_ and *ϕ*_0_, the vortices generated for other spectral components can be calculated by finding the locus in parameter space with condition $\sinh (2p({y}_{0}/{\omega }_{{\lambda }_{0}}))=2\pi {\varphi }_{0}$. The red, green and blue lines describe the locus of the single-charge vortices that can be generated for *λ* = 632.8 *nm* of He-Ne laser and *λ* = 514 *nm* and *λ* *=* 457 *nm* from Argon-ion laser with the same parameters that generated canonical vortex for the central wavelength of *λ*_0_ = 545 *nm* The slight asymmetry for the core of vortices generated for *λ* = 632.8 *nm* and *λ* = 457 *nm* compared to the symmetric core for central wavelength of *λ*_0_ = 545 *nm* can be inferred. In the realm of first-order optical modes comprising of Hermite-Gaussian and Laguerre-Gaussian modes that are represented on a modal/*Padgett* sphere^29, 30^ equivalent to the Poincaré sphere representation of the state of polarization, the stretching of the symmetric vortex core, for wavelengths neighbouring the central wavelength can be attributed to fractional orbital angular momentum (charge) in a way the fractional spin angular momentum is attributed to the elliptical state of polarization.

By scaling the lateral shear *y*_0_ introduced for different spectral components of the field such that *y*_0_(*λ*)=*y*_0_ *p*, by introducing dispersion inside the interferometer, it could be possible to maintain the single-charge vortex canonical for all the spectral components, if needed. In our experimental design using Sagnac interferometer, we preferred not to introduce dispersion for the lateral shear. For many practical applications such as the generation of high-power ultrashort vortex and vector pulses, this aspect can be safely ignored due to negligibly small asymmetry for the dark core in the vortices corresponding to spectral components spanning the visible spectrum as shown in Fig. 3.

### Interferometric generation of cylindrical vector beam from a scalar vortex beam

We briefly describe the method of generating a cylindrical vector beam from a scalar single-charge vortex beam. As shown in Fig. 6, at the output of the modified Mach-Zehnder interferometer, the quarter wave plate QWP introduced is oriented such that the states of polarization of the vortex beams with topological charge *l* = +1 and −1 are transformed to left (${\sigma }^{-}$) and right (${\sigma }^{+}$) circular states, respectively. Their on-axis, out-of-phase superposition generating an azimuthally polarized vector beam can be written in Jones matrix formalism as

9 $$[\begin{array}{c}{E}_{x}^{azim}\\ {E}_{y}^{azim}\end{array}]=\frac{1}{2}\exp (-i\phi )[\begin{array}{c}1\\ i\end{array}]-\frac{1}{2}\exp (i\phi )[\begin{array}{c}1\\ -i\end{array}]=i[\begin{array}{c}-\sin \,\phi \\ \,\cos \,\phi \end{array}]$$

The out-of-phase superposition can be attributed to the choice of the output port of the interferometer.

At the introduction of half-wave plate HWP_2_ following QWP, with its fast-axis oriented at an angle *θ*_2_ with respect to *x* axis, the transformation of the azimuthally-polarized optical field represented in Eq. (9) can be obtained by a rotation operation performed through R(*θ*) defined as

10 $$R(\theta )=[\begin{array}{c}\cos \,2\theta \,\,\,\sin \,2\theta \\ \sin \,2\theta -\,\cos \,2\theta \end{array}]$$

11 $$[\begin{array}{c}{E}_{x}^{hwp2}\\ {E}_{y}^{hwp2}\end{array}]=R({\theta }_{2})[\begin{array}{c}{E}_{x}^{azim}\\ {E}_{y}^{azim}\end{array}]=-i[\begin{array}{c}\sin (\phi -2{\theta }_{2})\\ \cos \,(\phi -2{\theta }_{2})\end{array}]$$

For *θ*_2_ = *π*/4, the optical field distribution represented in Eq. (11) describes a hybrid polarized optical field given by

12 $$[\begin{array}{c}{E}_{x}^{hybrid}\\ {E}_{y}^{hybrid}\end{array}]=R({\theta }_{2}=\pi /4)[\begin{array}{c}{E}_{x}^{azim}\\ {E}_{y}^{azim}\end{array}]=-i[\begin{array}{c}\sin (\phi -\pi /2)\\ \cos (\phi -\pi /2)\end{array}]$$

At the introduction of half-wave plate HWP_3_ following HWP_2_, with its fast-axis oriented at an angle *θ*_3_ with respect to *x* axis, the transformed optical field can be written as

13 $$[\begin{array}{c}{E}_{x}^{hwp3}\\ {E}_{y}^{hwp3}\end{array}]=R({\theta }_{3})R({\theta }_{2})[\begin{array}{c}{E}_{x}^{azim}\\ {E}_{y}^{azim}\end{array}]=i[\begin{array}{c}-\sin (\phi -2{\theta }_{2}+2{\theta }_{3})\\ \,\,\,\cos (\phi -2{\theta }_{2}+2{\theta }_{3})\end{array}]$$

For *θ*_2_ = *π*/4 and *θ*_3_ = *π*/2, the optical field distribution represented in Eq. (13) describes a radially polarized optical field given by

14 $$[\begin{array}{c}{E}_{x}^{rad}\\ {E}_{y}^{rad}\end{array}]=R({\theta }_{3}=\pi /2)R({\theta }_{2}=\pi /4)[\begin{array}{c}{E}_{x}^{azim}\\ {E}_{y}^{azim}\end{array}]=i[\begin{array}{c}-\sin (\phi +\pi /2)\\ \,\,\cos (\phi +\pi /2)\end{array}]$$

The sinusoidal variation of *x* and *y* components of the cylindrical vector optical field as a function of azimuthal coordinate is observed through rotating analyser as described in Fig. 7 in order to confirm their generation experimentally.
